# Dispersal of Late Triassic clam shrimps across Pangea linking northwestern Gondwana and central Pangea rift basins

**DOI:** 10.1038/s41598-024-66015-y

**Published:** 2024-07-01

**Authors:** Carlos M. Alarcón, Carina E. Colombi, Oscar F. Gallego, Juan M. Drovandi, Mateo D. Monferran

**Affiliations:** 1 Consejo Nacional de Investigaciones Científicas y Técnicas (CONICET), Buenos Aires, Argentina; 2 Instituto y Museo de Ciencias Naturales, Universidad Nacional de San Juan, CIGEOBIO, CONICET, Av. España 400 (N), J5400DNQ San Juan, San Juan Argentina; 3 Grupo Paleontología y paleoambientes continentales fanerozoicos, Centro de Ecología Aplicada del Litoral, CECOAL, CONICET, Universidad Nacional del Nordeste, UNNE, Ruta Provincial Nº 5, Km 2,5, W3400 Corrientes, Argentina; 4 Geología Histórica-Paleoinvertebrados-Micropaleontología (Área Ciencias de la Tierra -Departamento de Biología), Facultad de Ciencias Exactas, Naturales y Agrimensura (FaCENA), Universidad Nacional del Nordeste (UNNE), Av. Libertad 4450, W3400 Corrientes, Argentina

**Keywords:** Geology, Palaeontology, Sedimentology, Tectonics

## Abstract

Clam shrimps are a group of freshwater crustaceans who prospered during the Late Triassic. They were abundant in lacustrine sedimentary records of continental basins distributed throughout Pangea during this time. However, they show significant taxonomic differences between the clamp shrimp faunas from the rift basins of central Pangea and the southern Gondwanan basins. In this contribution, we show new fossil clam shrimp assemblages from the lacustrine sedimentary successions of the Eastern Cordillera of Colombia (the Bocas and Montebel formations), providing information on the Late Triassic species that inhabited the northwestern Gondwana basins. This study demonstrates that the basins of northwestern Gondwana shared Norian clamp shrimp species with rift basins of central Pangea and differed in their faunas with the basins of the southern portion of Gondwana. In addition, the Late Triassic clam shrimps paleobiogeographic distribution reflects the dispersal of this fauna throughout fluvial-lacustrine environments established in the rift valleys along the central Pangea. Therefore, the rift valleys produced during the early fragmentation of central Pangea could have acted as corridors for dispersion. Simultaneously, rift valleys also provided paleobiogeographic barriers that isolated the central Pangea clam shrimp faunas from southern Gondwana.

## Introduction

The first stage of the breakup of the Pangea supercontinent was a continuous process that developed from the Ladinian to the Triassic-Jurassic boundary. As a result of rifting processes, a system of fissures crossed the central part of the supercontinent from the Caribbean to the Tethys^1–7^. This fragmentation resulted in the separation of Gondwana and Laurasia and the simultaneous fragmentation of Laurasia into North America and Eurasia^2,4,5^.

The best-known examples of this early fragmentation are the sedimentary records of the Germanic Basin in central Europe and the rift basins of the central Atlantic margins in the United States, Canada, and Morocco^3,8–12^ (rift basins of central Pangea). These basins were propitious areas for the establishment and accumulation of lacustrine and fluvial successions^8,13–16^.

The sedimentary record of the rift basins of central Pangea hosts diverse and abundant groups of clam shrimps, also known (with different systematic and phylogenetic meanings) as “conchostracans” or spinicaudatans^17–20^. These non-marine invertebrates show global distribution along Pangea during the Late Triassic, allowing them to be used as biostratigraphic markers^21–27^. However, for this period, the central Pangea basins share a clam shrimp fauna, which shows substantial taxonomic differences compared to the faunas of the southern Gondwana basins^23,28^ (Supplementary Table 1). The factors responsible for the isolation of clam shrimp faunas within a single continental mass remain under debate.

This study focuses on two fossil assemblages from the Upper Triassic successions in the Eastern Cordillera of Colombia (the Bocas and Montebel formations), presenting a novel association of clam shrimps with high-resolution biostratigraphy and providing information on the Late Triassic species that inhabited the northwestern Gondwana basins. The clamp shrimp faunas of the northwestern Gondwana units differ from those of the southern Gondwana basins. However, the northwestern Gondwana basins share clam shrimp faunal components with the rift basins of central Pangea. Combining the biostratigraphic and paleobiogeographic data provides additional insights into the spatial distribution of the clam shrimp fauna. This paleobiogeographic data reflects the early breakup of central Pangea, whith the dispersal of clam shrimps through fluvial-lacustrine environments formed in the rift valleys during the Norian.

### Geological setting

The Colombian basins in northern South America were located at low paleolatitudes (0º-5º N) at the western margin of Pangea during the Late Triassic^29^. At this paleolatitude, the western margin of Pangea was affected by intracontinental rifting, resulting in a series of extensional basins forming during the Late Triassic^30–35^. In the Sierra Nevada de Santa Marta (SNSM) regions and the Eastern Cordillera, the filling of the basins is represented by the Los Indios, Corual, Tiburón, Bocas, Palermo, and Montebel formations.

**Figure 1:**
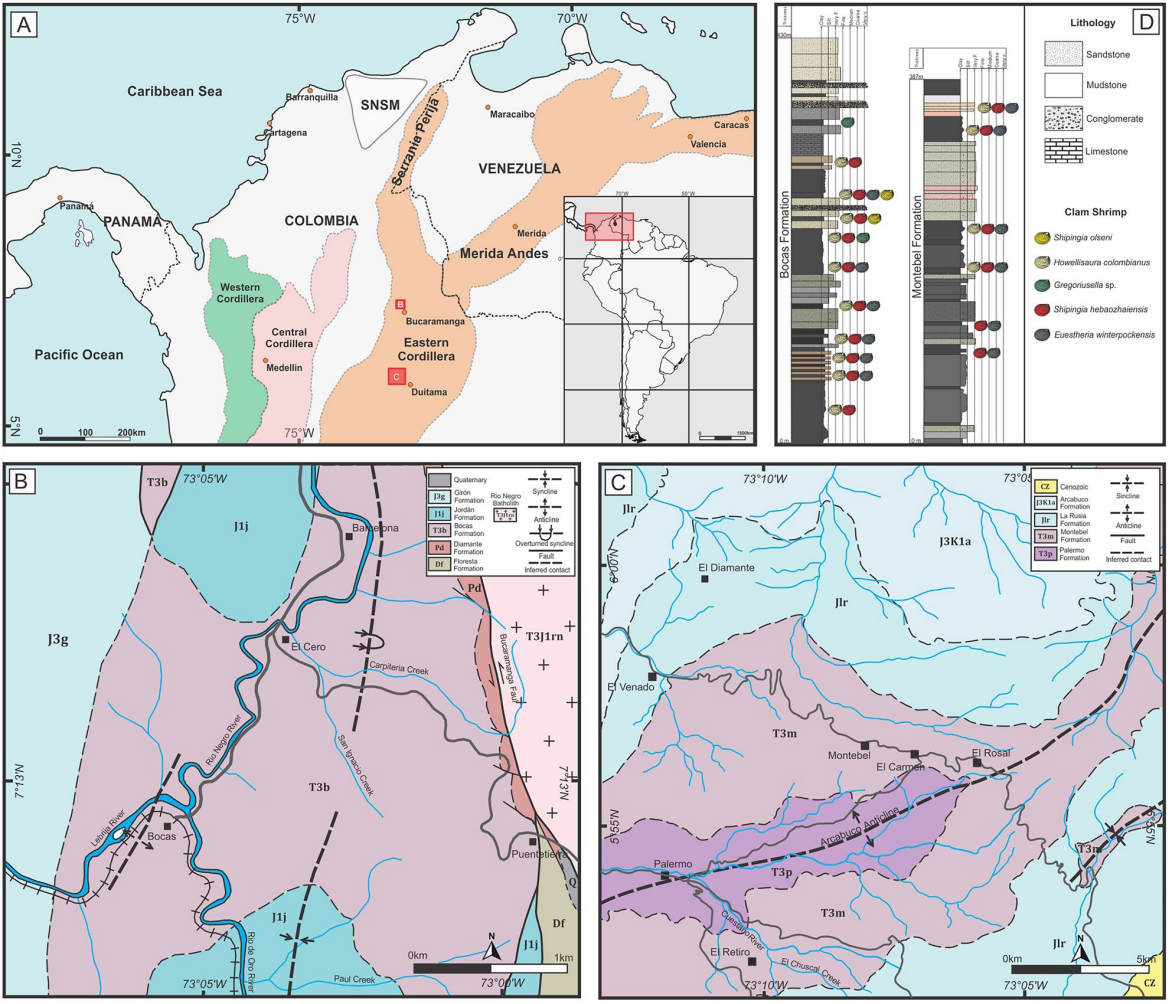
(**A**) The Eastern Cordillera of Colombia in northern South America. (**B**,**C**) Detail of the studied areas from the Bocas and Montebel formations. (**D**) Stratigraphic columns of the Bocas and Montebel formations showing the stratigraphic position of the studied clam shrimps. Maps drawn with QGIS 3.36 (https://www.qgis.org/en/site/index.html). Stratigraphic columns drawn with Inkscape 1.2.2 (https://inkscape.org/).

The Upper Triassic Bocas and Montebel formations are located in the Eastern Cordillera of Colombia (Fig. 1A–C). These units host fossil assemblages characterized by freshwater invertebrates (e.g. clam shrimps, darwinuloid ostracods, unionid bivalves), vertebrate teeth, fish scales, and plant remains^36–43^. The lithological and fossiliferous characteristics of the Bocas and Montebel formations indicate a lacustrine environment^38,42–44^.

## Results

### Clam shrimp assemblages

**Figure 2:**
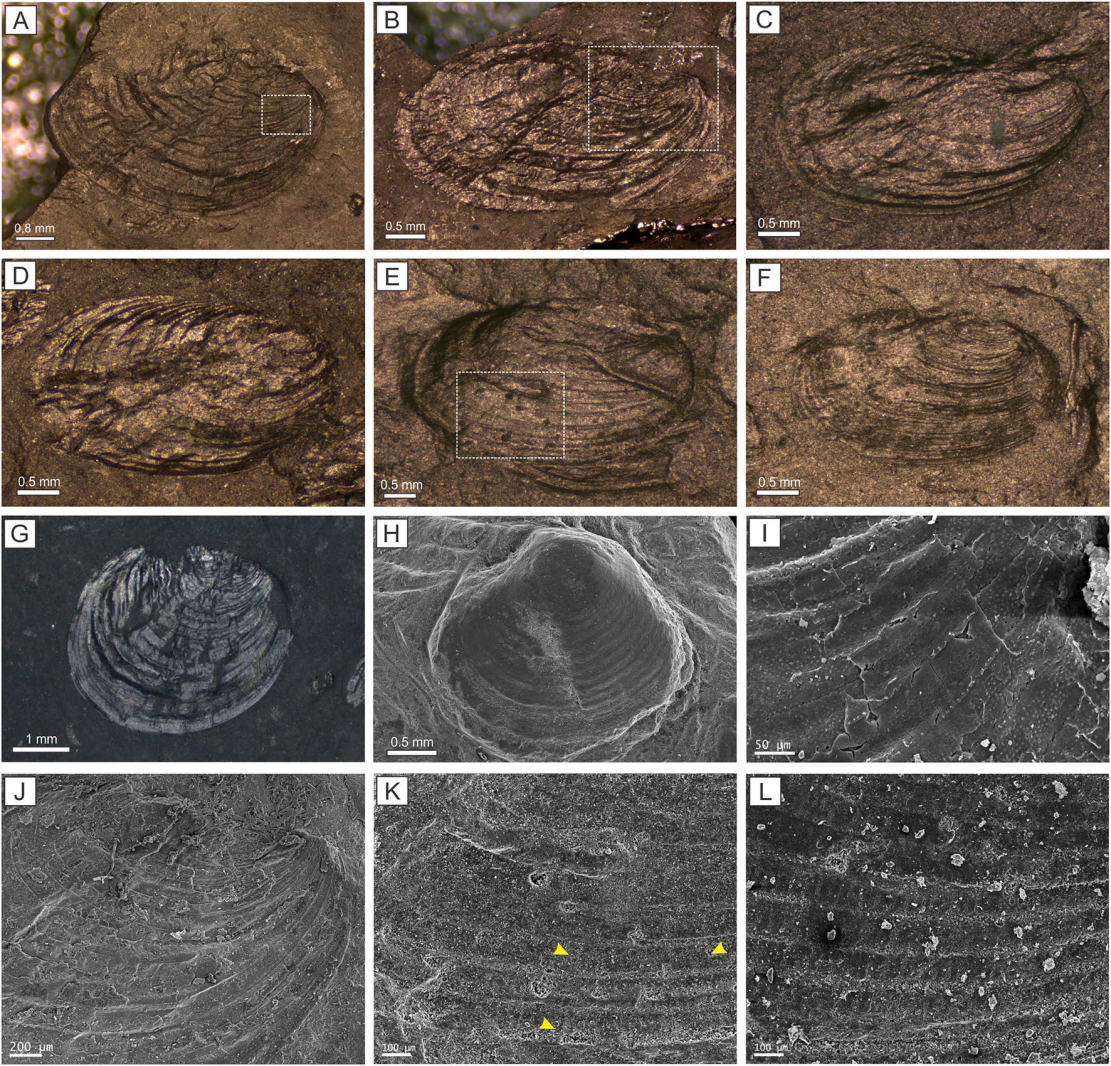
Fossil clam shrimps from the Upper Triassic of the Eastern Cordillera of Colombia. (**A**) *Shipingia olseni* Kozur and Weems, 2005. (**B**–**D**) *Shipingia hebaozhaiensis* Shen, 1976. (**E**,**F**) *Howellisaura colombianus* Bock, 1953. (**G)** *Euestheria winterpockensis* Bock, 1953. (**H**) *Gregoriusella* sp. (**I**) Detailed growth bands (white box in (**A**)) with pitted ornamentation of *Shipingia olseni*. (**J**) Detailed growth bands (white box in (**B**)) with smooth ornamentation of *Shipingia hebaozhaiensis*. (**K**) Detailed growth bands (white box in E) with radial lirae ornamentation of *Howellisaura colombianus.* The yellow arrows indicate the radial lirae. (**L**) Detail of the growth bands with radial lirae ornamentation of *Howellisaura colombianus.*

The identified clam shrimp assemblages exhibit significant species diversity and are abundant within the Bocas and Montebel formations (Fig. 1D; Supplementary systematic paleontology). The clam shrimp faunas in both units are characterized by an assemblage of species with the following components and percentage abundance: *Howellisaura colombianus* Bock, 1953 (51.5%), *Shipingia hebaozhaiensis* Shen, 1976 (28.5%), *Euestheria winterpockensis* Bock, 1953 (17.6%), *Gregoriusella* sp. (1.5%), and *Shipingia olseni* Kozur and Weems, 2005 (0.9%; Fig. 2).

### Biostratigraphy

The assemblage described from the Bocas and Montebel formations in Colombia allows for correlation with the *Shipingia hebaozhaiensis* zone defined in the Germanic Basin and the Newark Supergroup^22,23,25^. This correlation assigns a middle-late Alaunian age (middle Norian) to the Bocas and Montebel formations^22,23,25^. However, in the middle to upper part of the Bocas Formation, the occurrence of *Shipingia olseni*, a marker taxa of the Sevatian^21–23,25^, in association with *Shipingia hebaozhaiensis*, indicates the transition between the Alaunian and Sevatian for this specific interval. The overlap of *S. hebaozhaiensis* and *S. olseni* has also been reported in the Newark Supergroup, specifically in the Groveton Member of the Bull Run Formation in the Culpeper Basin^21,22^.

## Discussion and conclusions

### Paleobiogeography

**Figure 3:**
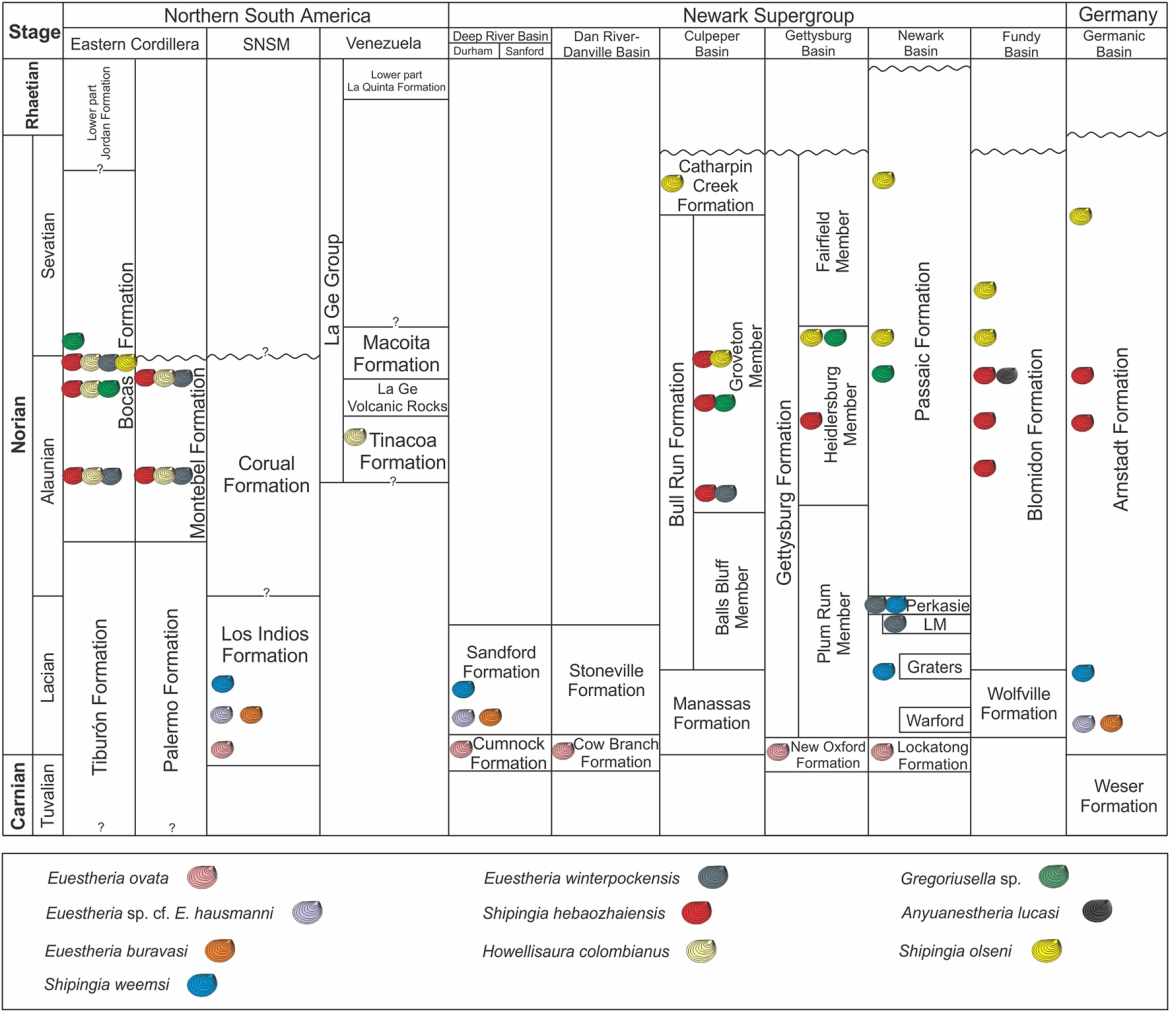
Correlation of stratigraphic units and common clam shrimp species of the Upper Triassic in the basins of northern South America and the rift basins of central Pangea. Species reports and unit ages are based on previous studies^12,21–25,34,45^. Figure drawn with Inkscape 1.2.2 (https://inkscape.org/).

The clam shrimp taxa described from the Bocas and Montebel formations in the Eastern Cordillera of Colombia (*Shipingia hebaozhaiensis*, *Euestheria winterpockensis*, *Gregoriusella* sp., *Shipingia olseni, and H. colombianus)* reveal common elements between the northwestern Gondwana basins and the rift basins of central Pangea. The common taxa (*Shipingia hebaozhaiensis, Euestheria winterpockensis, Gregoriusella sp., and Shipingia olseni*) have previously been reported in the Newark Supergroup for the Culpeper, Gettysburg, Fundy, and Newark basins (U.S.A. and Canada), as well as in the Germanic Basin (Germany). In addition, *H. colombianus* was reported in the Tinacoa Formation, along the Serranía del Perijá^45^ (Venezuela, South America). Therefore, this paleobiogeographical distribution would encompass the rift basins of central Pangea extending to the northwestern Gondwana basins during the middle-late Norian (middle Alaunian-early Sevatian; Fig. 3; Supplementary Table 2).

Nevertheless, older reports of clam shrimps preserved in the Los Indios Formation^34^ (the Sierra Nevada de Santa Marta, northern Colombia) provide evidence that the distribution of clamp shrimp assemblages in northwestern Gondwana and rift basins of central Pangea could have begun earlier, probably during the Lacian (early Norian). This early distribution is supported by *Euestheria ovata* Lea, 1856, *Euestheria* cf. *E*. *hausmanni* Schmidt, 1938, *Euestheria buravasi* Kobayashi, 1975 and *Shipingia weemsi* Kozur et al., 2012 present in the Los Indios Formation as well as in units of the Newark Supergroup and the Germanic Basin (Fig. 3; Supplementary Table 2).

### Distribution and dispersion of clam shrimps across rift valleys

**Figure 4:**
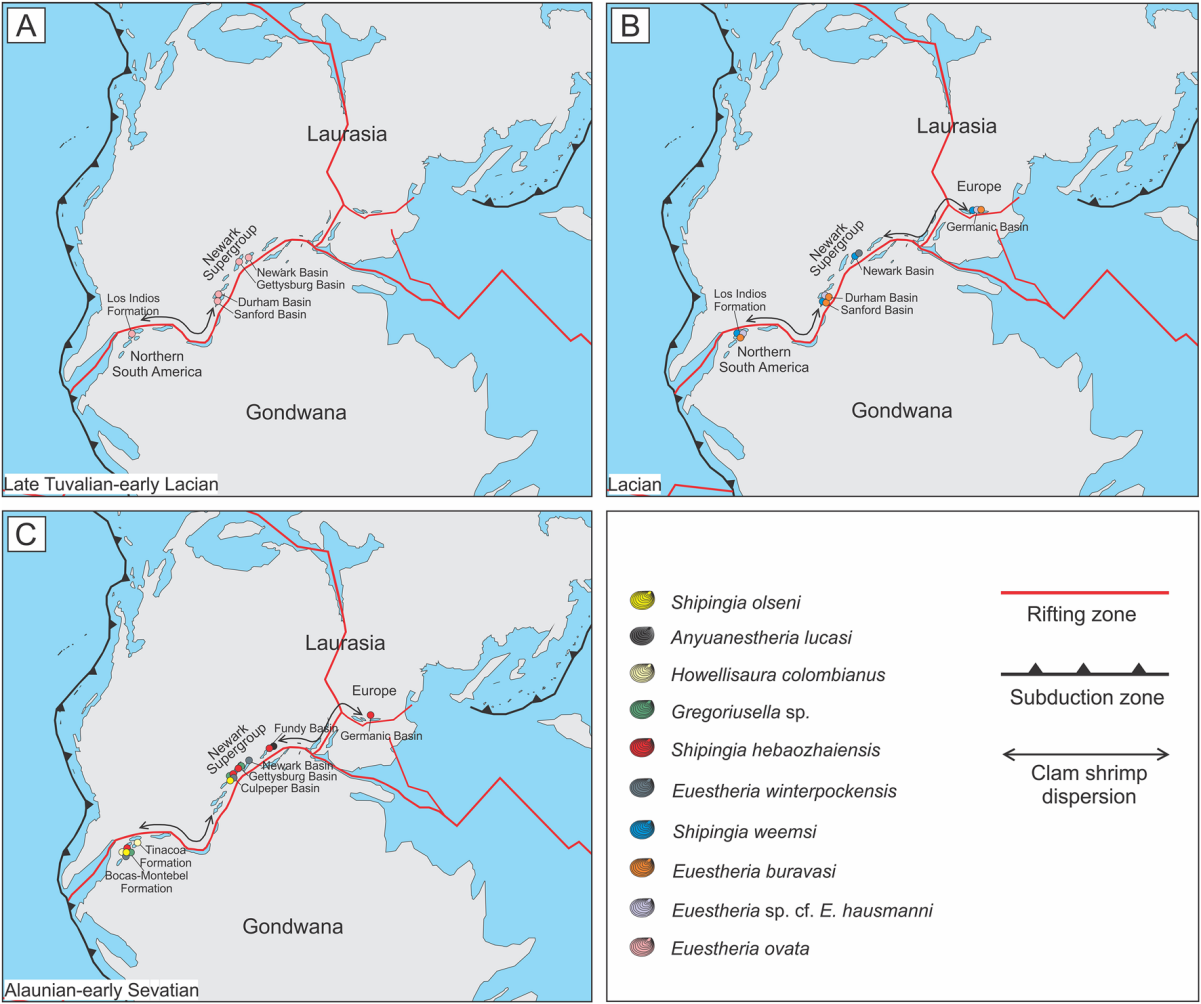
Paleobiogeographic distribution of common clam shrimp species from the Upper Triassic of the basins of northern South America and the rift basins of central Pangea. (**A**) Late Tuvalian-early Lacian. (**B**) Lacian. (**C**) Alaunian-early Sevatian. The red lines represent the rifting zones of central Pangea during the Late Triassic^2,5,6^. The scheme was developed based on paleogeographic reconstructions for the Late Triassic^57,58^. Figure drawn with Inkscape 1.2.2 (https://inkscape.org/).

The paleobiogeographic distribution of clam shrimps throughout central Pangea demonstrates that this fauna (i.e., *Euestheria ovata*, *Euestheria* cf. *E*. *hausmanni*, *Euestheria buravasi*, *Shipingia weemsi, Shipingia hebaozhaiensis*, *Euestheria winterpockensis*, *Gregoriusella* sp., *Shipingia olseni, H. colombianus)* extended across central Europe, the eastern margin of North America (Newark Supergroup), and northern South America during the Norian. This paleobiogeographic distribution coincides with the rift zones associated with the Late Triassic early fragmentation of central Pangea (Fig. 4).

During the latter, the fractured areas and related subsidences led to the formation of large rift valleys and a network of lakes and aligned river systems^46^. An analogous tectonic scenario can currently be observed in the Great Rift Valley of East Africa, resulting from the fracturing of the African Plate. The tectonic depressions are occupied by elongated and narrow lakes and river systems, reflecting the rift morphology that extends regionally (> 3000 km) from Mozambique to Ethiopia^47,48^.

The freshwater environments, supplied by a network of fluvial and lacustrine systems in the rift valleys that developed in a large continentalized Pangea may have provided uniquely favorable habitats for the establishment of the clamp shrimp faunas during the Late Triassic. Additionally, the clamp shrimp reproductive adaptations (i.e. accelerated biological cycle, dioecious reproduction, and the presence of abundant eggs capable of withstanding desiccation^28,49–54^) could have favored their high abundance in these habitats and the survival resistance of their eggs during transport across rift valleys.

This conditioned dispersion of clam shrimp fauna throughout the central Pangea basins associated with the early fragmentation (rift basins of the central Atlantic margins; Germanic Basin; northwestern Gondwana) could explain the taxonomic difference with the southern Gondwanan clamp shrimp faunas (Supplementary Table 1). Therefore, the rift valleys of central Pangea could have acted as a paleobiogeographic barrier favoring the isolation of these independently evolving faunas (provincialism).

## Methods

A total of 330 remarkably well-preserved specimens were collected from the Bocas and Montebel formations for systematic identification. These individuals were obtained from dark lacustrine claystones and siltstones (Fig. 1D). The specimens were observed with a binocular stereo microscope Leica S9D and photographed with a Leica Flexcam C1 camera. The S.E.M. (scanning electron microscope) images were obtained with a JEOL 5800LV microscope at the Secretaría General de Ciencia y Técnica, Universidad Nacional del Nordeste (UNNE). The previously proposed methodology was followed for taxonomic identification and biostratigraphic schemes^18,23,25,55,56^. All specimens are housed in the Instituto y Museo de Ciencias Naturales collection, Universidad Nacional de San Juan (registered numbers PISJ 111–117).

### Supplementary Information

Supplementary Information.

## Data Availability

No datasets were generated or analysed during the current study.
