# Supplementary material for: Dispersal of Late Triassic clam shrimps across Pangea linking northwestern Gondwana and central Pangea rift basins
Source: Sci Rep. 2024 Jul 1;14:15025. doi: 10.1038/s41598-024-66015-y (PMC11217500; doi:10.1038/s41598-024-66015-y)
Supplement: Supplementary file 1 — Supplementary Information. [file 41598_2024_66015_MOESM1_ESM.docx]

**Supplementary Information**

1. Supplementary Table(s)

**Table 1.**

| **CENTRAL PANGEA** | | | | | | | | | |  |
| --- | --- | --- | --- | --- | --- | --- | --- | --- | --- | --- |
|  |  |  |  |  |  |  |  |  |  |  |
| **Newark Supergroup** | | | | | | | | | |  |
| **Suborder** | **Superfamily** | **Family** | **Subfamily** | **Genus** | **Species** | **Author, year** | **Age/ Stratigraphic unit** | **Geographic location** | **Reference** |  |
| Spinicaudata | Eosestherioidea | Shipingiidae |  | *Laxitextella* | sp. cf. *L. laxitexta* | (Sandberger, 1871) Kozur, 1982 | Late Triassic (Carnian) / New Oxford Fm. - Newark Supergroup | Eastern USA | Kozur & Weems [1] |  |
|  |  |  |  | *Laxitextella* | *multireticulata* | (Reible, 1962) Kozur, 1982 | Late Triassic (Carnian) / New Oxford Fm; Falling Creek Fm. - Newark Supergroup | Eastern USA | Kozur & Weems [1] |  |
|  |  |  |  | *Shipingia* | *mcdonaldi* | Kozur & Weems, 2010 | Late Triassic (Norian) / Gettysburg Fm; Bull Run Fm. - Newark Supergroup | Eastern USA | Kozur & Weems [2]; Weems & Lucas [3] |  |
|  |  |  |  | *Shipingia* | *weemsi* | Kozur et al., 2013 | Late Triassic (Norian)/ Sanford Fm; Passaic Fm. - Newark Supergroup | Eastern USA | Weems & Lucas [3] |  |
|  |  |  |  | *Shipingia* | *hebaozhaiensis* | Shen (in Zhang et al., 1976) | Late Triassic (Norian) / Bull Run Fm; Gettysburg Fm; Blomidon Fm - Newark Supergroup | Eastern USA and southeastern Canada | Kozur & Weems [1]; Weems & Lucas [3]; Kozur & Weems [4] |  |
|  |  |  |  | *Shipingia* | *olseni* | Kozur & Weems, 2005 | Late Triassic (Norian) / Bull Run Fm; Gettysburg Fm; Catharpin Creek Fm; Blomidon Fm - Newark Supergroup | Eastern USA and southeastern Canada | Kozur & Weems [1]; Kozur & Weems [2]; Weems & Lucas [3]; Kozur & Weems [4] |  |
|  |  |  |  | *Redondestheria* | *grovetonensis* | Kozur & Weems, 2005 | Late Triassic (Norian) / Bull Run Fm; Gettysburg Fm; Blomidon Fm - Newark Supergroup | Eastern USA and southeastern Canada | Kozur & Weems [4]; Weems & Lucas [3] |  |
|  |  |  |  | *Anyuanestheria* | sp. | Zhang & Chen, 1976 | Late Triassic (Norian) / Cow Branch Fm. - Newark Supergroup | Eastern USA | Kozur & Weems [1] |  |
|  |  |  |  | *Anyuanestheria* | *lucasi* | Kozur & Weems, 2011 | Late Triassic (Norian) / Blomidon Fm. - Newark Supergroup | Southeastern Canada | Weems & Lucas [3]; Kozur & Weems [5] |  |
|  |  | Family Uncertain |  | *Wannerestheria* | *pennsylvanica* | (Wanner 1926) Kozur & Weems, 2010 | Late Triassic (Carnian-Norian) / Gettysburg Fm; Lockatong Fm. - Newark Supergroup | Eastern USA | Kozur & Weems [2] |  |
|  |  | Euestheriidae |  | *Gregoriusella* | sp. | Kozur & Weems, 2010 | Late Triassic (Norian) / Bull Run Fm; Gettysburg Fm; Passaic Fm - Newark Supergroup | Eastern USA | Weems & Lucas [3] |  |
|  |  |  |  | *Euestheria* | *princetonensis* | (Bock, 1953) Kozur & Weems, 2007 | Late Triassic (Carnian-Norian) / Cow Branch Fm; Cumnock Fm; Lockatong Fm. - Newark Supergroup | Eastern USA | Kozur & Weems [1]; Kozur & Weems [2] |  |
|  |  |  |  | *Euestheria* | *ovata* | (Lea, 1956) Kozur & Weems, 2007 | Late Triassic (Norian) / Lockatong Fm; New Oxford Fm; Cumnock Fm; Cow Branch Fm. - Newark Supergroup | Eastern USA | Kozur & Weems [1] |  |
|  |  |  |  | *Euestheria* | *hausmanni* | (Schmidt, 1938) Kozur & Weems, 2007 | Late Triassic (Norian) / Lockatong Fm; New Oxford Fm; Cumnock Fm. - Newark Supergroup | Eastern USA | Kozur & Weems [1] |  |
|  |  |  |  | *Euestheria* | sp. cf. *E. hausmanni* | (Schmidt, 1938) Kozur & Weems, 2007 | Late Triassic (Norian) / Triangle Brick Quarry, “lithofacies association II”. - Newark Supergroup | Eastern USA | Kozur & Weems [1] |  |
|  |  |  |  | *Euestheria* | *buravasi* | Kobayashi, 1975 | Late Triassic (Norian) / Triangle Brick Quarry, “lithofacies association II”; Cumnock Fm; Sanford Fm. - Newark Supergroup | Eastern USA | Kozur & Weems [1]; Kozur & Weems [2]; Weems & Lucas [3] |  |
|  |  |  |  | *Euestheria* | *winterpockensis* | (Bock, 1953) Kozur & Weems, 2007 | Late Triassic (Norian) / Tuckahoe Fm; Falling Creek Fm; Passaic Fm; Bull Run Fm. - Newark Supergroup | Eastern USA | Kozur & Weems [1]; Weems & Lucas [3] |  |
|  |  |  |  | *Euestheria* | *brodieana* | (Jones, 1862) Raymond, 1946 | Late Triassic (Rhaetian) / Midland Formation; Culpeper Basin - Newark Supergroup | Eastern USA | Kozur & Weems [1] |  |
| Leaina | Estherielloidea | Estheriellidae |  | *Acadiestheriella* | *cameroni* | Kozur & Weems, 2010 | Late Triassic (Norian) / Blomidon Fm. - Newark Supergroup | Southeastern Canada | Kozur & Weems [2] |  |
|  |  |  |  |  |  |  |  |  |  |  |
| **Europe** | | | | | | | | | |  |
| **Suborder** | **Superfamily** | **Family** | **Subfamily** | **Genus** | **Species** | **Author, year** | **Age/ Stratigraphic unit** | **Geographic location** | **Reference** |  |
| Spinicaudata | Eosestherioidea | Shipingiidae |  | *Laxitextella* | sp. cf. *L. laxitexta* | (Sandberger, 1871) Kozur, 1982 | Late Triassic (Carnian) / Middle Schilfsandstein | Germany | Kozur & Weems [1] |  |
|  |  |  |  | *Laxitextella* | *laxitexta* | (Sandberger, 1871) Kozur, 1982 | Late Triassic (Carnian) / Grabfeld Fm; lithostratigraphic unit, Upper Silesia | Germany and Poland | Geyer & Kelber [6] |  |
|  |  |  |  | *Laxitextella* | *freybergi* | Kelber & Kozur in Kozur & Weems, 2007 | Late Triassic (Carnian) / Hassberge Fm. | Germany | Geyer & Kelber [6] |  |
|  |  |  |  | *Laxitextella* | *multireticulata* | (Reible, 1962) Kozur, 1982 | Late Triassic (Carnian) / Grabfeld Fm. | Germany, Southern Alps, England | Kozur & Weems [1]; Geyer & Kelber [6] |  |
|  |  |  |  | *Laxitextella* | *dorsorecta* | (Reible, 1962) Seegis, 1997 | Late Triassic (Carnian) / Mainhardt Fm. | Germany | Geyer & Kelber [6] |  |
|  |  |  |  | *Laxitextella* | *seegisi* | Kozur in Kozur & Weems, 2007 | Late Triassic (Carnian) / Lehrberg Beds of southwestern Germany; Krasiejów locality in Opole Silesia | Germany and Poland | Kozur & Weems [1] |  |
|  |  |  |  | *Anyuanestheria* | sp. A | Kozur & Weems, 2007 | Late Triassic / Middle Schilfsandstein | Germany | Kozur & Weems [1] |  |
|  |  |  |  | *Shipingia* | *weemsi* | Kozur et al., 2012 | Late Triassic (Norian) / Arnstadt Fm. | Germany | Kozur et al. [7] |  |
|  |  |  |  | *Shipingia* | *mcdonaldi* | Kozur & Weems, 2010 | Late Triassic (Norian) / Arnstadt Fm. | Germany | Kozur & Weems [2] |  |
|  |  |  |  | *Shipingia* | *hebaozhaiensis* | Shen (in Zhang et al., 1976) | Late Triassic (Norian) / Arnstadt Fm. | Germany | Kozur & Weems [1] |  |
|  |  |  |  | *Shipingia* | *olseni* | Kozur & Weems, 2005 | Late Triassic (Norian) / Stubensandstein 3 of southern Germany | Germany | Kozur & Weems [1] |  |
|  |  |  |  | *Shipingia* | *gerbachmanni* | Hauschke & Kozur, 2011 | Late Triassic (Norian) / Lower Saxony | Germany | Hauschke & Kozur [8] |  |
|  |  | Palaeolimnadiopseidae | Asiolimnadiopseinae | *Eosolimnadiopsis* | *gallegoi* | Kozur in Kozur & Weems, 2007 | Late Triassic / Weser Fm. - Dolomie Beaumont Horizon | Germany, France | Kozur & Weems [1] |  |
|  |  | Euestheriidae |  | *Euestheria* | *kozuri* | Geyer & Kelber, 2017 | Late Triassic (Norian) / Hassberge Fm. | Germany | Geyer & Kelber [6] |  |
|  |  |  |  | *Euestheria*? | sp. |  | Late Triassic / Hassberge Fm. | Germany | Geyer & Kelber [6] |  |
|  |  |  |  | *Euestheria* | *multicostata* | (Geyer, 1987) Geyer & Kelber, 2017 | Late Triassic / Grabfeld Fm. | Germany | Geyer & Kelber [6] |  |
|  |  |  |  | *Euestheria* | *hausmanni* | (Schmidt, 1938) Kozur & Weems, 2007 | Late Triassic (Norian) / Coburg Sandstein of southern Germany | Germany | Kozur & Weems [1] |  |
|  |  |  |  | *Euestheria* | sp. cf. *E. hausmanni* | (Schmidt, 1938) Kozur & Weems, 2007 | Late Triassic (Norian) / Arnstadt Fm. | Germany | Kozur & Weems [1] |  |
|  |  |  |  | *Euestheria* | *buravasi* | Kobayashi, 1975 | Late Triassic (Norian) / Arnstadt Fm. | Germany | Kozur & Weems [2] |  |
|  |  |  |  | *Euestheria* | *winterpockensis* | (Bock, 1953) Kozur & Weems, 2007 | Late Triassic / Grabfeld Fm. | Germany | Kozur & Weems [1] |  |
|  |  |  |  | *Euestheria* | *brodieana* | (Jones, 1862) Raymond, 1946 | Late Triassic (Rhaetian) | Germany, France and England | Kozur & Weems [1] |  |
|  |  |  |  | *Gregoriusella* | *polonica* | Kozur, Niedźwiedzki & Sulej in Kozur & Weems, 2010 | Late Triassic (Rhaetian) / Exter Fm; Upper Silesia | Germany and Poland | Geyer & Kelber [6] |  |
|  |  |  |  | *Gregoriusella* | *striatula* | Geyer & Kelber, 2017 | Late Triassic / Hassberge Fm; Exter Fm. | Germany | Kozur & Weems [2]; Geyer & Kelber [6] |  |
|  |  |  |  |  |  |  |  |  |  |  |
| **Northern South America** | | | | | | | | | |  |
| **Suborder** | **Superfamily** | **Family** | **Subfamily** | **Genus** | **Species** | **Author, year** | **Age/ Stratigraphic unit** | **Geographic location** | **Reference** |  |
| Spinicaudata | Eosestherioidea | Euestheriidae |  | *Euestheria* | *ovata* | (Lea, 1956) Kozur & Weems, 2007 | Late Triassic (Norian) / Los Indios Fm. | Northern South America, Colombia | Gómez et al. [9] |  |
|  |  |  |  | *Euestheria* | sp. cf. *E. hausmanni* | (Schmidt, 1938) Kozur & Weems, 2007 | Late Triassic (Norian) / Los Indios Fm. | Northern South America, Colombia | Gómez et al. [9] |  |
|  |  |  |  | *Euestheria* | *buravasi* | Kobayashi, 1975 | Late Triassic (Norian) / Los Indios Fm. | Northern South America, Colombia | Gómez et al. [9] |  |
|  |  |  |  | *Euestheria* | *winterpockensis* | (Bock, 1953) Kozur & Weems, 2007 | Late Triassic (Norian) / Bocas Fm; Montebel Fm. | Northern South America, Colombia | This work |  |
|  |  |  |  | *Gregoriusella* | sp. |  | Late Triassic (Norian) / Bocas Fm. | Northern South America, Colombia | This work |  |
|  |  | Fushunograptiidae |  | *Howellisaura* | *colombianus* | Bock, 1953 | Late Triassic (Norian) / Bocas Fm; Montebel Fm; Tinacoa Fm. | Northern South America, Colombia | Bock [10]; Odreman & Benedetto [11]; This work |  |
|  |  | Shipingiidae |  | *Shipingia* | *weemsi* | Kozur et al., 2012 | Late Triassic (Norian) / Los Indios Fm. | Northern South America, Colombia | Gómez et al. [9] |  |
|  |  |  |  | *Shipingia* | *hebaozhaiensis* | Shen (in Zhang et al., 1976) | Late Triassic (Norian) / Bocas Fm; Montebel Fm. | Northern South America, Colombia | This work |  |
|  |  |  |  | *Shipingia* | *olseni* | Kozur & Weems, 2005 | Late Triassic (Norian) / Bocas Fm. | Northern South America, Colombia | This work |  |
|  |  |  |  |  |  |  |  |  |  |  |
| **Northern Africa** | | | | | | | | | |  |
| **Suborder** | **Superfamily** | **Family** | **Subfamily** | **Genus** | **Species** | **Author, year** | **Age/ Stratigraphic unit** | **Geographic location** | **Reference** |  |
|  |  |  |  |  |  |  |  |  |  |  |
| Spinicaudata | Eosestherioidea | Shipingiidae |  | *Laxitextella* | *laxitexta* | (Sandberger, 1871) Kozur, 1982 | Late Triassic (Carnian) / Timezgadiouine Fm. | North Africa, Morocco | Tourani et al. [12] |  |
|  |  |  |  | *Laxitextella* | *multireticulata* | (Reible, 1962) Kozur, 1982 | Late Triassic (Carnian / Timezgadiouine Fm. | North Africa, Morocco | Tourani et al. [12] |  |
|  |  |  |  | *Shipingia* | *gerbachmanni* | Hauschke & Kozur, 2011 | Late Triassic (Norian) / Bigoudine Fm. | North Africa, Morocco | Weems & Lucas [3] |  |
|  |  | Euestheriidae |  | *Euestheria* | *minuta* | (von Zieten, 1833) Raymond, 1946 | Late Triassic / Timezgadiouine Fm; north Constantine, Sid-Al-bou-Krizi | North Africa, Morocco and Algerie | Tourani et al. [12]; Tasch [13] |  |
|  |  |  |  | *Euestheria* | *princetonensis* | (Bock, 1953) Kozur & Weems, 2007 | Late Triassic / Timezgadiouine Fm. | North Africa, Morocco | Tourani et al. [12] |  |
|  |  |  |  | *Euestheria* | *forbesi* | (Jones, 1862) Raymond, 1946 | Late Triassic / north Constantine, Sid-Al-bou-Krizi | Algeria - Africa. | Tasch [13]; Defretin et al. [14] |  |
|  |  |  |  | *Euestheria* | sp. |  | Late Triassic / Timezgadiouine Fm. | North Africa, Morocco | Tourani et al. [12] |  |
|  |  |  |  | *Gregoriusella* | *polonica* | Kozur, Niedźwiedzki & Sulej in Kozur & Weems, 2010 | Late Triassic (Rhaetian) / Bigoudine Fm. | North Africa, Morocco | Weems & Lucas [3] |  |
|  |  |  |  | *Gregoriusella* | sp. |  | Late Triassic / Timezgadiouine Fm. | North Africa, Morocco | Tourani et al. [12] |  |
|  |  |  |  | Genus indet. |  |  |  |  |  |  |
|  |  |  |  | *"Estheria"* | *destombesi* | (Defretin, 1950) Cuvellier et al., 2015 | Late Triassic / Argana-Bigoudine, Haute Atlas. | North Africa, Morocco | Defretin & Fauvelete [15] |  |
|  |  |  |  |  |  |  |  |  |  |  |
| **SOUTHERN GONDWANA** | | | | | | | | | |  |
|  |  |  |  |  |  |  |  |  |  |  |
| **Africa** | | | | | | | | | |  |
| **Suborder** | **Superfamily** | **Family** | **Subfamily** | **Genus** | **Species** | **Author, year** | **Age/ Stratigraphic unit** | **Geographic location** | **Reference** |  |
| Spinicaudata | Vertexioidea | Paleolimnadiidae | Estheriininae | *Palaeolimnadia (Palaeolimnadia)* | cf. *wianamattensis* | (Mitchell, 1927) Tasch, 1987 | Late Triassic / Cassanje III Series Phyllopod Beds Stage | R. Angola - Africa | Tasch [13] |  |
|  |  |  |  | *Palaeolimnadia (Grandilimnadia)* | *oesterleni* | Tasch, 1987 | Late Triassic / Cassanje I Series Phyllopod Beds Stage | R. Angola - Africa | Tasch [13] |  |
|  |  |  |  | *Palaeolimnadia (Grandilimnadia)* | *africania* | Tasch, 1987 | Late Triassic / Cassanje I Series Phyllopod Beds Stage | R. Angola - Africa | Tasch [13] |  |
|  |  |  |  | *Palaeolimnadia (Grandilimnadia)* | sp. |  | Late Triassic / Cave Sandstone | K. Lesotho - Africa | Tasch [16] |  |
|  |  |  |  | *Estheriina (Nudusia)* | cf. *rewanensis* | Tasch (Tasch & Jones, 1979) | Late Triassic / Cassanje III Series Phyllopod Beds Stage | R. Angola - Africa | Tasch & Jones [17] |  |
|  |  |  |  | *Afrolimnadia* | *sibiriensis* | Tasch, 1987 | Late Triassic / Cave Sandstone | R. South Africa - Africa. | Tasch [13] |  |
|  |  | Palaeolimnadiopseidae | Palaeolimnadiopseinae | *Palaeolimnadiopsis* | *lubefuensis* | Defretin-Lefranc, 1967 | Late Triassic or Jurassic (pre Oxfordian) / Haute Lueki Series | D.R. Congo - Africa. | Defretin-Lefranc [18] |  |
|  |  |  |  | *Pteriograpta* | *reali* | (Teixeira, 1958) Teixeira, 1961 | Late Triassic or Jurassic / Cassanje III | R. Angola- Africa. | Tasch [13] |  |
|  |  | Lioestheriidae | Vertexiinae | *Echinestheria* | *marimbensis* | Marlière, 1950 | Late Triassic or Jurassic (pre Oxfordian) / Haute Lueki Series - Late Triassic / Cassanje III. | D.R. Congo / R. Angola - Africa | Tasch [13] |  |
|  |  |  |  | *Cornia* | *angolata* | Tasch, 1987 | Late Triassic / Cassanje I Series Phyllopod Beds Stage | R. Angola - Africa | Tasch [13] |  |
|  |  |  |  | *Cornia* | *haughtoni* | Tasch, 1984 | Late Triassic / Cave Sandstone | K. Lesotho - Africa | Tasch [16] |  |
|  | Eosestherioidea | Euestheriidae |  | *Euestheria* | *stockleyi* | Tasch, 1984 | Late Triassic / Cave Sandstone | K. Lesotho - Africa | Tasch [16], |  |
|  |  |  |  | *Euestheria* | *thabaningensis* | Tasch, 1987 | Late Triassic / Cave Sandstone | K. Lesotho - Africa | Tasch [13] |  |
|  |  | Orthothemosiidae |  | *Glyptoasmussia* | *luekiensis* | Defretin-Lefranc, 1967 | Late Triassic or Jurassic (pre Oxfordian) / Haute Lueki Series. | D.R. Congo - Africa. | Defretin-Lefranc [18] |  |
|  |  |  |  | *Pseudestheria* | *lepersonnei* | Defretin-Lefranc, 1967 | Late Triassic - Late Cretaceous /Cassanje III - Kwango Series - l´Inzia beds. | D.R. Congo / R. Angola - Africa. | Defretin-Lefranc [18] |  |
|  |  |  |  | *Orthothemos* | *draperi* | (Jones & Woodward, 1894) Raymond, 1946 | Late Triassic / Shale overlying Cave Sandstone | R. South Africa - Africa. | Jones & Woodward [19] |  |
|  |  | Eosestheriidae |  | *Carapacestheria* | *malangensis* | (Marlière, 1950) Everman, 2007 | Late Triassic or Jurassic (pre-Oxfordian) Haute Lueki Series / Cassanje III. | R. Angola - D.R. Congo - Africa | Marlière [20] |  |
|  | Estheriteoidea | Asmusiidae |  | *Asmussia* | *loockii* | Tasch, 1984 | Late Triassic / Cave Sandstone | K. Lesotho - Africa | Tasch [16] |  |
|  |  | Fushunograptidae |  | *"Bairdestheria"* | *kitariensis* | Defretin-Lefranc, 1967 | Late Triassic or Jurassic - Late Cretaceous / Cassanje III - Lubilash System - Kwango Series, Canyon d l'Inzia. | R. Angola - D.R. Congo - Africa. | Defretin-Lefranc [18] |  |
|  |  | “Estheriteoidean indet” |  | *"Lioestheria"* | *cassambensis* | Teixeira, 1960 | Late Triassic ? / Cassanje III ?. | R. Angola - Africa. | Teixeira [21] |  |
|  |  |  |  | *"Lioestheria"* | *lesothoensis* | Tasch, 1984 | Late Triassic / Cave Sandstone | K. Lesotho / R. South Africa - Africa | Tasch [16] |  |
| Estheriellina | Afrograptioidea | Afrograptidae |  | *Congestheriella* | *lualabensis* | (Leriche, 1913) Kobayashi, 1954 | Late Triassic - Jurassic / Lualaluba beds | D.R. Congo - Africa. | Leriche [22] |  |
|  | Estheriellioidea | Estheriellidae |  | *Estheriella* | *bornhardti* | Janensch, 1925 (in Tasch, 1987) | Late Triassic / Hatambulo Beds, Beaufort Series. | U.R. Tanzania - Africa. | Tasch [13] |  |
|  |  |  |  | *Estheriella* | *moutai* | (Leriche, 1932) Defretin-Lefranc, 1967 | Late Triassic or Jurassic (pre Oxfordian) / Haute Lueki Series - Upper Triassic / Cassanje III. | D.R. Congo / R. Angola - Africa. | Leriche [23] |  |
|  |  |  |  | *Estheriella?* | sp. | Dietrich, 1939 (in Tasch, 1987) | Triassic / Nord Adamawa | R. Cameroon - Africa. | Tasch [16] |  |
|  |  |  |  |  |  |  |  |  |  |  |
| **Argentina - Chile** | | | | | | | | | |  |
| **Suborder** | **Superfamily** | **Family** | **Subfamily** | **Genus** | **Species** | **Author, year** | **Age/Stratigraphic unit** | **Geographical location** | **Reference** |  |
| Spinicaudata | Vertexioidea | ?Paleolimnadiidae |  | *"Pseudoestheria"* | *leonense* | Rusconi, 1946 | Middle-Late Triassic / Potrerillos Fm. | Mendoza - Argentina | Rusconi [24] |  |
|  |  |  |  | *"Pseudoestheria"* | *contorta* | Rusconi, 1948 | Middle-Late Triassic / Potrerillos Fm. | Mendoza - Argentina | Rusconi [25] |  |
|  |  |  |  | *"Pseudoestheria"* | *minoprioi* | Rusconi, 1947 | Middle-Late Triassic / Potrerillos Fm. | Mendoza - Argentina | Rusconi [26] |  |
|  |  | Paleolimnadiopseidae | Asiolimnadiopseinae | *Endolimnadiopsis* | *rusconii* | Gallego, 2005 | Middle-Late Triassic /Agua de la Zorra Fm. | Mendoza - Argentina | Gallego [27] |  |
|  |  | Pemphilimnadiopseidae |  | *Challaolimnadiopsis* | *mendozaensis* | Shen and Gallego (in Shen et al., 2001) Tassi & Gallego, (in Tassi et al., 2015) | Middle-Late Triassic / Potrerillos Fm. | Mendoza - Argentina | Shen et al. [28] |  |
|  |  | Lioestheriidae | Vertexiinae | *Cornia* | *falconeri* | Gallego et al., 1993 (Tassi, 2016) | Middle-Late Triassic / Potrerillos Fm. | Mendoza - Argentina/ Dpto. de Rivera, Uruguay | Tassi [29] |  |
|  | Eosestherioidea | Ulugkemiidae |  | *Triasulugkemia* | *ischichucaensis* | Gallego (Gallego & Melchor, 2000) | Late Triassic / Ischichuca Fm. | La Rioja - Argentina | Gallego & Melchor [30] |  |
|  |  |  |  | *Triasulugkemia* | *shenyanbini* | Gallego (Gallego & Melchor, 2000) | Late Triassic / Cacheuta Fm. | Mendoza - Argentina | Gallego & Melchor [30] |  |
|  |  |  |  | *Triasulugkemia* | sp. |  | Late Triassic / Cacheuta Fm. | Mendoza - Argentina | Tassi [29] |  |
|  |  | Euestheriidae |  | *Euestheria* | *forbesi* | (Jones) Raymond, 1946 | Late Triassic / Los Rastros Fm; Ischichuca Fm; Cacheuta Fm; Potrerillos Fm; Cerro de las Cabras Fm. | La Rioja/San Juan/Mendoza - Argentina. | Gallego [31]; Gallego [32] |  |
|  |  |  |  | *Euestheria* | *mangaliensis* | (Jones) Raymond, 1946 | Late Triassic / Cacheuta Fm; Potrerillos Fm. | Mendoza - Argentina | Gallego [31]; Gallego [32] |  |
|  |  |  |  | *Euestheria* | *menendezi* | Gallego, 1999 -Gallego & Tassi (in Tassi et al., 2015) | Middle-Late Triassic / Potrerillos Fm; Cerro de las Cabras Fm. | Mendoza - Argentina | Gallego [32] |  |
|  |  |  |  | *Euestheria* | sp. 1 |  | Late Triassic / Cacheuta Fm. | Mendoza - Argentina | Gallego [32] |  |
|  |  |  |  | *Euestheria* | sp. 2 |  | Late Triassic / Los Rastros Fm. | La Rioja/San Juan - Argentina. | Gallego [32] |  |
|  |  |  |  | *Euestheria* | sp. 3 |  | Late Triassic / Los Rastros Fm. | La Rioja/San Juan - Argentina. | Gallego [32] |  |
|  |  |  |  | *Euestheria* | sp. 4 |  | Late Triassic / Cacheuta Fm. | Mendoza - Argentina | Gallego [32] |  |
|  |  |  |  | *Euestheria* | sp. 5 |  | Late Triassic / Ischichuca Fm. | La Rioja - Argentina. | Gallego [32] |  |
|  |  | Loxomegaglyptidae |  | *Triasoglypta* | sp. 1 (=Triasoglypta sp. 2) |  | Late Triassic / Cacheuta Fm; Potrerillos Fm. | Mendoza - Argentina | Gallego [32] |  |
|  |  |  |  | *Triasoglypta* | sp. 2 |  | Late Triassic / Los Rastros Fm. | La Rioja/San Juan - Argentina. | Gallego [32] |  |
|  |  |  |  | *Triasoglypta* | sp. 3 |  | Late Triassic / Cacheuta Fm; Potrerillos Fm. | Mendoza - Argentina | Gallego [32] |  |
|  |  |  |  | *Triasoglypta* | sp. 4 |  | Late Triassic / Casa de Piedra Fm. | San Juan -Argentina | Gallego [32] |  |
|  |  |  |  | *Triasoglypta* | sp. A |  | Late Triassic / Ischigualasto Fm. | San Juan -Argentina | Gallego [32] |  |
|  |  |  |  | *Eoparaleptestheria* | *triasiana* | Gallego, 1999 | Late Triassic / Cacheuta Fm. | Mendoza - Argentina | Gallego [32] |  |
|  |  | Eosestheriidae |  | *Menucoestheria* | *puquenensis* | Gallego (in Gallego & Covacevich, 1998) | Late Triassic / ?Profeta Fm; ?Pichidangui Fm. | Chile | Gallego & Covacevich [33] |  |
|  |  |  |  | *Menucoestheria* | *terneraensis* | Gallego (in Gallego & Covacevich, 1998) | Late Triassic / La Ternera Fm. - Santa Juana Fm. | Chile | Gallego & Covacevich [33] |  |
|  |  |  |  | *Menucoestheria* | *wichmanni* | Gallego, 2010 | Late Triassic / Vera Fm; Potrerillos Fm. | Rio Negro/ Mendoza - Argentina | Gallego [34] |  |
|  | Estheriteoidea | Polygraptidae |  | *Dendrostracus* | sp. |  | Late Triassic / Ischigualasto Fm. | San Juan - Argentina | Tassi [29] |  |
|  |  |  |  | *Polygrapta* | *troncosoi* | (Gallego) Gallego et al., 2005 | Late Triassic / La Ternera Fm; Santa Juana Fm. | Chile | Gallego et al. [27]; Gallego & Covacevich [33] |  |
|  |  |  |  | Genus Unknown |  |  |  |  |  |  |
|  |  |  |  | *"Lioestheria"* | *striolatissima* | (Rusconi) Tasch, 1987 | Late Triassic / Cacheuta Fm. | Mendoza - Argentina | Gallego [32] |  |
|  |  |  |  |  |  |  |  |  |  |  |
| **Brazil** | | | | | | | | | |  |
| **Suborder** | **Superfamily** | **Family** | **Subfamily** | **Genus** | **Species** | **Author, year** | **Age / Stratigraphic unit** | **Geographic location** | **Reference** |  |
| Spinicaudata | Vertexioidea | Paleolimnadiidae | Estheriininae | *Palaeolimnadia (Grandilimnadia)* | *glenleensis* | (Mitchell, 1927) Katoo, 1971 | Late Triassic / Santa Maria Fm. | Rio Grande do Sul-Brazil | Katoo [35] |  |
|  |  |  |  | *Palaeolimnadia (Palaeolimnadia)* | *wianamattensis* | (Mitchell, 1927) Pinto, 1956 | Late Triassic / Santa Maria Fm. | Rio Grande do Sul-Brazil | Pinto [36] |  |
|  |  |  |  | *Palaeolimnadia?* | cf. *wianamattensis* | (Mitchell, 1927) Katoo, 1971 (in Tasch, 1987) | Late Triassic / Santa Maria Fm. | Rio Grande do Sul-Brazil | Katoo [35] |  |
|  |  |  |  | *Estheriina (Nudusia)* | sp. | Pinto, 1956 | Late Triassic / Santa Maria Fm. | Rio Grande do Sul-Brazil | Pinto [36] |  |
|  |  | Palaeolimnadiopseidae | Palaeolimnadiopseinae | *Palaeolimnadiopsis* | sp. 2 |  | Late Triassic /Santa Maria Fm. | Rio Grande do Sul-Brazil | Tasch [13] |  |
|  |  | Lioestheriidae | Vertexiinae | *?Echinestheria* | sp. |  | Late Triassic / Botucatú Fm. | Mato Grosso - Brazil | Katoo [35] |  |
|  |  |  |  | *Echinopalma* | *semigibosa* | Cardoso, 1962 | Late Triassic / Motuca Fm. | Piaui- Brazil | Cardoso [37] |  |
|  | Eosestherioidea | Euestheriidae |  | *Euestheria* | *azambujai* | Pinto 1956 | Late Triassic /Santa Maria Fm. | Rio Grande do Sul-Brazil | Pinto [36] |  |
|  |  |  |  | *Euestheria* | *cf. emmonsi* | (Raymond, 1946) Katoo, 1971 | Late Triassic / Posto Castelinho | Rio Grande do Sul-Brazil | Katoo [35] |  |
|  |  |  |  | *Pseudoasmussia* | sp. A |  | Late Triassic / Santa Maria Fm. | Rio Grande do Sul - Brazil | Katoo [35] |  |
|  |  |  |  | *Pseudoasmussia*? | sp. B |  | Late Triassic / Santa Maria Fm. | Rio Grande do Sul-Brazil | Katoo [35] |  |
|  |  | Orthothemosiidae? |  | *Orthothemos?* | sp. |  | Late Triassic /Santa Maria Fm. | Rio Grande do Sul-Brazil | Katoo [35] |  |
|  |  |  |  | *Pseudoasmussiata* | *katooae* | Tasch, 1987 | Late Triassic /Santa Maria Fm. | Rio Grande do Sul-Brazil | Tasch [13] |  |
|  |  | Loxomegaglyptidae |  | *Triasoglypta* | *santamariensis* | (Gallego, 1996) Gallego, 1999 | Late Triassic / Santa Maria Fm. | Rio Grande do Sul-Brazil | Gallego [38] |  |
|  |  |  |  | Genus Unknown |  |  |  |  |  |  |
|  | Estheriteoidea | Fushunograptidae |  | *"Bairdestheria"* | *mendesi* | Almeida, 1950 | Late Triassic - Early Cretaceous/Piramboia, Massacará-São Sebastião Botucatú Fm | São Paulo-Brazil | Almeida [39] |  |
|  |  |  |  | *"Lioestheria"* | *florianensis* | Cardoso, 1962 | Late Triassic / Motuca Fm. | Piaui- Brazil | Cardoso [37] |  |
|  |  |  |  | *"Lioestheria"* | sp. |  | Late Triassic / Santa Maria Fm. | Rio Grande do Sul-Brazil | Katoo [35] |  |
|  |  |  |  |  |  |  |  |  |  |  |
| **India** | | | | | | | | | |  |
| **Suborder** | **Superfamily** | **Family** | **Subfamily** | **Genus** | **Species** | **Author, year** | **Age/Stratigraphic unit** | **Geographical location** | **Reference** |  |
| Spinicaudata | Vertexioidea | Paleolimnadiidae | Estheriininae | *Palaeolimnadia (Palaeolimnadia)* | *kamthiensis* | Ghosh, 2011 | Triassic / Kamthi Fm. | Orissa - India | Ghosh [40] |  |
|  |  |  |  | *Palaeolimnadia (Grandilimnadia)* | *geei* | Ghosh, 2011 | Triassic / Parsora Fm. | India | Ghosh [40] |  |
|  |  | Palaeolimnadiopseidae | Palaeolimnadiopseinae | *Palaeolimnadiopsis* | *dhauraiensis* | Ghosh, 2011 | Triassic / Parsora Fm. | Madhya Pradesh - India | Ghosh [40] |  |
|  |  |  |  | *Palaeolimnadiopsis* | *gond* | Ghosh, 2011 | Triassic | Madhya Pradesh - India | Ghosh [40] |  |
|  |  |  |  | *Palaeolimnadiopsis* | *odissi* | Ghosh, 2011 | Triassic / Parsora Fm. | Orissa - India | Ghosh [40] |  |
|  |  |  |  | *Macrolimnadiopsis* | *mitrai* | Ghosh, 2011 | Triassic / Kamthi Fm. | Madhya Pradesh - India | Ghosh [40] |  |
|  |  | Lioestheriidae | Vertexiinae | *Cornia* | *shahi* | Ghosh, 2011 | Triassic / Mangli Bed - Pachmarhi Fm. | Maharashtra - India | Ghosh [40] |  |
|  |  |  |  | *Cornia* | *sonensis* | Ghosh, 2011 | Triassic / Pachmarhi - Panchet Fm. | Madhya Pradesh - India | Ghosh [40] |  |
|  |  |  |  | *Curvacornutus* | *mahadev* | Ghosh, 2011 | Triassic / Pachmarhi Fm. | Madhya Pradesh - India | Ghosh [40] |  |
|  |  |  |  | *Curvacornutus* | *dhupgarhensis* | Ghosh, 2011 | Triassic / Pachmarhi Fm. | Madhya Pradesh - India | Ghosh [40] |  |
|  |  |  |  | *Echinestheria* | *satpuraensis* | Ghosh, 2011 | Triassic / Pachmarhi Fm. | Madhya Pradesh - India | Ghosh [40] |  |
|  |  |  |  | *Gabonestheria* | *gopali* | Ghosh, 2011 | Triassic / Kamthi Fm. | West Bengal - India | Ghosh [40] |  |
|  |  |  |  | *Indomonocarina* | *chandrai* | Ghosh, 2011 | Triassic / Pachmarhi Fm. | Madhya Pradesh - India | Ghosh [40] |  |
| Estheriellina | Estheriellioidae | Estheriellidae |  | *Estheriella (Lioestheriata)* | *mukherjii* | Ghosh, 2011 | Triassic / Kamthi Fm. | Orissa - India | Ghosh [40] |  |
|  |  |  |  | *Estheriella (Lioestheriata)* | *rathini* | Ghosh, 2011 | Triassic / Parsora Fm. | India | Ghosh [40] |  |
|  |  |  |  | *Estheriella (Pachmariella)* | *tuberculatus* | Ghosh, 2011 | Triassic / Pachmari Fm. | Madhya Pradesh - india | Ghosh [40] |  |
|  |  |  |  | *Cornutestheriella* | sp. |  | Triassic / Pachmarhi Formation | Madhya Pradesh-India | Ghosh [40] |  |
|  |  |  |  |  |  |  |  |  |  |  |
| **Australia** | | | | | | | | | |  |
| **Suborder** | **Superfamily** | **Family** | **Subfamily** | **Genus** | **Species** | **Author, year** | **Age/Stratigraphic unit** | **Geographical location** | **Reference** |  |
| Spinicaudata | Vertexioidea | Paleolimnadiidae | Estheriininae | *Palaeolimnadia (Grandilimnadia)* | *glenleensis* | (Mitchell, 1927) Tasch, 1987 | Late Triassic / Wianamatta Group | Glenlee Homestead, New South Wales | Mitchell [41] |  |
|  |  |  |  | *Palaeolimnadia (Grandilimnadia)* | sp. |  | Late Triassic / Wianamatta Group | Denmark Hill / Ipswich, Queensland | Tasch [13] |  |
|  |  |  |  | *Palaeolimnadia (Palaeolimnadia)* | *wianamattensis* | (Mitchell, 1927) Tasch, 1979 | Late Triassic / Wianamatta Group | Glenlee Homestead, New South Wales | Mitchell [41] |  |
|  |  | Lioestheriidae | Vertexiinae | *Cornia* | *coghlani* | (Etheridge, Jr., 1888) Tasch 1979, in Tasch & Jones, 1979 | Late Triassic / Wianamatta Group | Glenlee Homestead, New South Wales | Tasch [13] |  |
|  | Eosestherioidea | Euestheriidae |  | *Euestheria* | *ipsviciensis* | (Mitchell, 1927) Kobayashi, 1945 | Late Triassic / Wianamatta Group | Denmark Hill / Ipswich, Queensland | Tasch [13] |  |
|  |  | Family Unknown |  | Genus Unknown |  |  |  |  |  |  |
|  |  |  |  | *Cyzicus* | cf. *mangliensis?* |  | Late Triassic / Wianamatta Group | Denmark Hill / Ipswich, Queensland | Tasch [13] |  |
|  | Estheriteoidea | “Estheriteoidean indet” |  | *"Lioestheria"* | *australensis* | (Novojilov, 1958) Tasch in Tasch & Jones, 1979 | Late Triassic / Wianamatta Group | Denmark Hill / Ipswich, Queensland | Tasch [13] |  |

**Table 1|** Clam shrimps from the Late Triassic of central Pangea and southern Gondwana.

**Table 2.**

| **Taxa** | **Geographic location** | **Basin** | **Unit** | **Reference** |
| --- | --- | --- | --- | --- |
| *Euestheria ovata* | USA Newark Supergroup | Gettysburg Basin | New Oxford Formation | Kozur & Weems [1] |
|  | USA Newark Supergroup | Deep River Basin | Cumnock Formation | Kozur & Weems [1] |
|  | USA Newark Supergroup | Deep River Basin | Upper Cow Branch Formation | Kozur & Weems [1] |
|  | USA Newark Supergroup | Newark Basin | Lower Lockatong Formation | Kozur & Weems [1]; Kozur & Weems [2] |
|  | Northern South America |  | Lower Los Indios Formation | Gómez et al. [9] |
| *Euestheria sp. cf. E. hausmanni* | USA Newark Supergroup | Deep River Basin | Lower Sanford Formation | Kozur & Weems [1] |
|  | Germany | Germanic Basin | Lowermost Arnstadt Formation | Kozur & Weems [1] |
|  | Northern South America |  | Los Indios Formation | Gómez et al. [9] |
| *Euestheria buravasi* | USA Newark Supergroup | Deep River Basin | Sanford Formation | Kozur & Weems [1]; Kozur & Weems [2]; Weems & Lucas [3] |
|  | Germany | Germanic Basin | Lower Arnstadt Formation | Kozur & Weems [2] |
|  | Northern South America |  | Los Indios Formation | Gómez et al. [9] |
| *Shipingia weemsi* | USA Newark Supergroup | Deep River Basin | Sanford Formation | Weems & Lucas [3] |
|  | USA Newark Supergroup | Newark Basin | Passaic Formation | Weems & Lucas [3] |
|  | Germany | Germanic Basin | Lower Arnstadt Formation | Kozur et al. [7] |
|  | Northern South America |  | Los Indios Formation | Gómez et al. [9] |
| *Euestheria winterpockensis* | USA Newark Supergroup | Newark Basin | Passaic Formation | Weems & Lucas [3] |
|  | USA Newark Supergroup | Culpeper Basin | Bull Run Formation, Groveton Member | Weems & Lucas [3] |
|  | Northern South America |  | Bocas Formation | This work |
|  | Northern South America |  | Montebel Formation | This work |
| *Shipingia hebaozhaiensis* | USA Newark Supergroup | Culpeper Basin | Bull Run Formation, Groveton Member | Kozur & Weems [4]; Weems & Lucas [3] |
|  | Canada Newark Supergroup | Fundy Basin | Blomidon Formation | Weems & Lucas [3] |
|  | USA Newark Supergroup | Gettysburg Basin | Gettysburg Formation, Heidlesrsburg Member | Kozur & Weems [1] |
|  | Germany | Germanic Basin | Lower Arnstadt Formation | Kozur & Weems [1] |
|  | Northern South America |  | Bocas Formation | This work |
|  | Northern South America |  | Montebel Formation | This work |
| *Gregoriusella sp.* | USA Newark Supergroup | Newark Basin | Passaic Formation | Weems & Lucas [3] |
|  | USA Newark Supergroup | Gettysburg Basin | Gettysburg Formation, Heidlesrsburg Member | Weems & Lucas [3] |
|  | USA Newark Supergroup | Culpeper Basin | Bull Run Formation, Groveton Member | Weems & Lucas [3] |
|  | Northern South America |  | Bocas Formation | This work |
| *Howellisaura colombianus* | Northern South America |  | Bocas Formation | This work |
|  | Northern South America |  | Montebel Formation | Bock [10]; This work |
|  | Northern South America |  | Tinacoa Formation | Odreman & Benedetto [11] |
| *Shipingia olseni* | USA Newark Supergroup | Newark Basin | Passaic Formation | Kozur & Weems [4]; Kozur & Weems [1] |
|  | USA Newark Supergroup | Culpeper Basin | Bull Run Formation, Groveton Member | Kozur & Weems [4]; Kozur & Weems [1]; Weems & Lucas [3] |
|  | Canada Newark Supergroup | Fundy Basin | Blomidon Formation | Kozur & Weems [1] |
|  | USA Newark Supergroup | Gettysburg Basin | Gettysburg Formation, Heidlesrsburg Member | Weems & Lucas [3] |
|  | Germany | Germanic Basin | Arnstadt Formation | Kozur & Weems [4]; Kozur & Weems [1] |
|  | Northern South America |  | Bocas Formation | This work |

**Table 2|** Common species of Late Triassic clam shrimps from the rift basins of central Pangea and northwestern Gondwana.

1. Supplementary Information

**Systematic paleontology**

C**lass Branchiopoda Latreille, 1817**

**Subclass Phyllopoda Preuss, 1951**

**Order Diplostraca Gerstaecker, 1866**

**Suborder Onychocaudata Olesen & Richter 2013**

**Infraorder Spinicaudata Linder, 1945**

**Superfamily Eosestherioidea Zhang & Chen, in Zhang et al., 1976**

**Family Shipingiidae Kozur and Weems, 2005**

**Genus *Shipingia* Shen, in Zhang et al., 1976**

**Type species:** *Shipingia* *hebaozhaiensis*Shen, in Zhang et al., 1976

***Shipingia* *hebaozhaiensis* Shen, in Zhang et al., 1976**

**Comments:** The studied material includes 94 specimens. Of these, 75 are from the middle part of the Bocas Formation, near Bucaramanga, Colombia, and 19 are from the lower and upper part of the Montebel Formation, near Palermo, Paipa, Colombia. Two morphotypes were identified based on the carapace shape. Morphotype 1 has an elongated oval outline with a longer dorsal margin. Morphotype 2 is oval in shape and taller than 1, with a shorter dorsal margin. Carapace valve size ranges from small to very large.  Morphotype 1, L= 1.4 - 5.5 mm, H= 0.8 - 2.6 mm.  Morphotype 2, L= 1.2 - 6.3 mm, H= 0.9 - 3.6 mm. The morphological characteristics of these specimens are consistent with the diagnosis and descriptions^3,4^.

***Shipingia* *olseni* Kozur & Weems, 2005**

**Comments:** The studied material consists of 3 specimens from the middle part of the Bocas Formation, type locality of the Bocas Formation, near Bucaramanga, Colombia. The specimens are oval in shape. Valve sizes range from large to very large. The dorsal margin is straight and long. The umbo is located in the anterior zone. The ornamentation between growth lines is pitted. Measurements, L= 4.2 - 5.9 mm and H= 2.9 - 3.6 mm. The morphological characteristics of these specimens are consistent with the original diagnosis and descriptions^3,4^

**Family *Euestheriidae* Defretin-Lefranc, 1965**

**Genus *Euestheria* Depéret & Mazeran*,* 1912**

**Type species:** *Euestheria minuta* Von Zieten

***Euestheria winterpockensis* (Bock) Kozur & Weems, 2007**

**Comments:** The studied material includes 58 specimens. Of these, 48 are from the middle part of the Bocas Formation, near Bucaramanga, Colombia, and 10 are from the lower and upper part of the Montebel Formation, near Palermo, Paipa, Colombia. Two morphotypes are identified. Morphotype 1 has a rounded shape and Morphotype 2 has a subrounded shape. The size of the carapace valve varies from very small to large.  Morphotype 1, L= 1.1 - 4.3 mm, H= 0.9 - 3.7 mm.  Morphotype 2, L= 1.1 - 3.1 mm, H= 0.9 - 2.6 mm. The morphological characteristics of these specimens agree with the original diagnosis and descriptions^2,10^.

**Genus *Gregorisuella*, Kozur & Weems, 2010**

**Type species:** *Gregoriusella polonica Kozur, Niedźwiedzki & Sulej* in Kozur and Weems, 2010

***Gregoriusella* sp.**

**Comments:** The studied material consists of 5 specimens. Of these, 3 are from the middle part of the Bocas Formation, near Bucaramanga, Colombia, and 2 are from the Bocas Formation, Aguachica, Cesar, Colombia. Carapace valve with oval shape. The size of the carapace valve varies from small to very small.  Measurements, L= 1.6 - 2.4 mm and H= 1.1 - 1.9 mm. The morphological characteristics of these specimens are consistent with the original descriptions^2,3^.

**Superfamily Estheriteoidea Zhang and Chen, in Zhang et al., 1976**

**Family Fushunograptidae Wang in Hong et al., 1974**

**Genus *Howellisaura* Bock 1953**

**Type species**: *Howellites colombianus* Bock (1953)

***Howellisaura colombianus* (Bock) Bock, 1953**

**Comments:** The studied material includes 170 specimens. Of these, 132 are from the middle part of the Bocas Formation, near Bucaramanga, Colombia, and 38 are from the lower and upper part of the Montebel Formation near Palermo, Paipa, Colombia. Two morphotypes were identified based on carapace shape. Morphotype 1 has an elongated oval shape with a longer dorsal margin. Morphotype 2 is oval in shape with a shorter dorsal margin. Carapace valve size ranges from very small to very large. The ornamentation is consistent of radial lirae. Measurements, Morphotype 1, L= 1.1 - 5.6 mm, H= 0.5 - 3.1 mm.  Morphotype 2 L= 0.9 - 7.8 mm and H= 0.6 - 4.8 mm. The morphological characteristics of these specimens agree with the original diagnosis and descriptions^10,42^.

**References**

1. Kozur, H. W. & Weems, R. E. Upper Triassic conchostracan biostratigraphy of the continental basins of eastern North America: its importance for correlating Newark Supergroup events with the Germanic Basin and the International Geologic Time Scale. in *The Global Triassic. New Mexico Museum of Natural History and Science Bulletin* (eds. Lucas, S. G. & Spielmann, J. A.) vol. 41 137–188 (2007).

2. Kozur, H. W. & Weems, R. E. The biostratigraphic importance of conchostracans in the continental Triassic of the northern hemisphere. *Geological Society, London, Special Publications* **334**, 315–417 (2010).

3. Weems, R. & Lucas, S. A revision of the Norian Conchostracan Zonation in North America and its implications for Late Triassic North American tectonic history. in *Fossil Record 4* (eds. Sullivan, R. & Lucas, S.) 303–317 (New Mexico Museum of Natural History and Science Bulletin 67., 2015).

4. Kozur, H. W. & Weems, R. E. Conchostracan evidence for a late Rhaetian to early Hettangian age for the CAMP volcanic event in the Newark Supergroup, and a Sevatian (late Norian) age for the immediately underlying beds. *Hallesches Jahrbuch Geowissenschaft* **27**, 21–51 (2005).

5. Kozur, H. W. & Weems, R. E. Additions to the uppermost Alaunian through Rahetian (Triassic) conchostracan zonation of North America. in *New Mexico Museum of Natural History and Science Bulletin* vol. 53 295–300 (2011).

6. Geyer, G. & Kelber, K.-P. Spinicaudata (“Conchostraca,” Crustacea) from the Middle Keuper (Upper Triassic) of the southern Germanic Basin, with a review of Carnian–Norian taxa and suggested biozones. *PalZ* **92**, 1–34 (2018).

7. Kozur, H. W., Franz, M. & Bachmann, G. Shipingia weemsi n.sp., a biostratigraphically important conchostracan species from the uppermost Carnian and lowermost Norian of Central Europe. in *Triassic Geology and Paleontology* (eds. Tanner, L. H., Spielmann, J. A. & Lucas, S. G.) 1–6 (New Mexico Museum of Natural History and Science, Bulletin, 2012).

8. Hauschke, N. & Kozur, H. Two new conchostracan species from the Late Triassic of the Fuchsberg, northern foreland of the Harz Mountains northeast of Seinstedt (Lower Saxony, Germany). in *Fossil Record 3* vol. 53 187–194 (New Mexico Museum of Natural History and Science, Bulletin, 2011).

9. Gómez, C., Kammer, A., Bernet, M., Piraquive, A. & von Quadt, A. Late Triassic rift tectonics at the northernmost Andean margin (Sierra Nevada de Santa Marta). *J South Am Earth Sci* **105**, 102953 (2021).

10. Bock, W. American Triassic estherids. *J Paleontol* **27**, 62–76 (1953).

11. Odreman, O. & Benedetto, G. Paleontología y edad de la Formación Tinacoa, Sierra de Perijá, Venezuela. in *Memorias del V Congreso Geológico de Venezuela* vol. 1 15–32 (1977).

12. Tourani, A. *et al.* Characterization of the Carnian Pluvial Episode in the Argana Basin (Western High Atlas, Morocco): An approach based on sedimentology, clay mineralogy and paleosols. *Palaeogeogr Palaeoclimatol Palaeoecol* **627**, 111720 (2023).

13. Tasch, P. *Fossil Conchostraca of the Southern Hemisphere and Continental Drift: Paleontology, Biostratigraphy, and Dispersal*. (Geological Society of America, 1987).

14. Defretin, S., Delga, M. D. & Lambert, A. Faunules du Trias Supérieur dans le Nord-Constantinois (Algérie). *Bulletin Société d’Histoire Naturelle l’Afrique du Nord* **44**, 185–195 (1953).

15. Defretin, S. & Fauvelet, E. Présence de Phyllopodes triasiques dans la région d’Argana-Bigoudine (Haut-Atlas occidental). *Notes et Mémoires du service géologique du Maroc* **85**, 129–134 (1951).

16. Tasch, P. Biostratigraphy and paleontology of some conchostracan-bearing beds in southern Africa. *Paleontologia Africania* **15**, 61–85 (1984).

17. Tasch, P. & Jones, P. Carboniferous and Triassic Conchostraca from the Canning Basin, Western Australia. *Australia Bureau of Mineral Resources, Geological and Geophysical Bulletin* **185**, 3–20 (1979).

18. Defretin-Lefranc, S. Étude sur les phyllopodes du Bassin du Congo. *Annales du Musee Royal de l’Afrique Central, Sciences Geologique* **56**, 1–122 (1967).

19. Jones, R. T. & Woodward, H. On some fossil Phyllopoda. *Geological Magazine* **1**, 289–294 (1894).

20. Marlière, R. Ostracodes et Phyllopodes du Systeme du Karroo au Congo Beige et les regions avoisinantes: Tervuren, Belgique, Musee du Congo Beige. *Annales Sciences Geologiques* **8**, 7–43 (1950).

21. Teixeira, C. Sur quelques fossiles du Karrov de Ia Lunda, Angola: Angola, Museo do Dundo. 97 Preprint at (1960).

22. Leriche, M. Entomostraces des couches du Lualaba (Congo-Beige). *Revue Zoologique Africaine* **3**, 3–6 (1913).

23. Leriche, M. Sur les premiers fossiles découverts, au nord de l’Angola, dans le prolongement des couches du Lubilash, et sur le synchronisme des couches du Lubilash et des couches de Lualaba. *Academic des Sciences de Paris, Comptes Rendus* **195**, 395–400 (1932).

24. Rusconi, C. Nuevos peces triásicos de El Challao, Mendoza. *Revista de la Sociedad de  Historia y Geografía de Cuyo* **1**, 1–15 (1946).

25. Rusconi, C. Apuntes sobre el Triásico y el Ordovícico de El Challao, Mendoza. . *Revista  del Museo Historia Natural* **2**, 165–198 (1948).

26. Rusconi, C. Acerca de Estheria minoprioi (Ostracoda) de Mendoza. *Boletin de la  Facultad de Ciencias Físicas y Naturales* **9**, 753–758 (1947).

27. Gallego, O. F. First record of the family Palaeolimnadiopseidae Defretin-Le Franc, 1965 (Crustacea–Conchostraca) in the Triassic of Argentina. *J South Am Earth Sci* **18**, 223–231 (2005).

28. Shen, Y. B., Gallego, O. & Zavattieri, A. M. A new conchostracan genus form from the Triassic Potrerillos Formation, Argentina. *Acta Geologica Leopoldensia* **24**, 227–236 (2001).

29. Tassi, L. V. La diversidad de las faunas de invertebrados continentales y sus cambios durante el Pérmico y Triásico de la Argentina. (Universidad Nacional de Córdoba, Córdoba , 2016).

30. Gallego, O. F. & Melchor, R. N. La familia Ulugkemiidae Novozhilov, 1958 (Conchostraca) en el Triásico de la Argentina: Implicancias paleobiogeográficas. *Ameghiniana* **37**, 47–58 (2000).

31. Gallego, O. F. Conchostracos triásicos de Mendoza y San Juan,Argentina. *Ameghiniana* **29**, 159–175 (1992).

32. Gallego, O. F. Estudio sistemático de las faunas de conchostracos triásicos de la República Argentina. (Universidad Nacional de Córdoba, Córdoba, 1999).

33. Gallego, O. F. & Covacevich, V. Conchostracos triásicos delas Regiones de Antofagasta, Atacama y Coquimbo, Chile. *Revista Geológica de Chile* **25**, 115–139 (1998).

34. Gallego, O. F. A new crustacean clam shrimp (Spinicaudata: Eosestheriidae) from the Upper Triassic of Argentina and its importance for ‘conchostracan’ taxonomy. *Alcheringa* **34**, 179–195 (2010).

35. Katoo, Y. Conchostráceos Mesozóicos do Sul do Brasil: Contribuçâo á Estratigrafia das Formaçôes Santa Maria e Botucatú. (Universidad Federal de Rio Grande do Sul., Rio Grande do Sul, 1971).

36. Pinto, I. D. Arthropódos da Formaçâo Santa Maria (Triássico Superior) do Río Grande do Sul, com notícias sôbre alguns restos vegetais. *Boletim Sociedade Brasileira de Geologia* **5**, 75–87 (1956).

37. Cardoso, R. N. Alguns conchostráceos mesozóicos do Brasil. *Escola Federal de Minas de Ouro Petro* **11**, 43–76 (1962).

38. Gallego, O. F. Triasoglypta santamariensis Gallego nov. comb. (Conchostraca) de la Formación Santa María (Triásico Medio-Superior) de Brasil. *Revista Universidade Guarulhos, Serie Geociências* **IV**, 61–66 (1999).

39. Almeida, F. Uma fáunula de crustáceos bivalvos do Arenito Botucatu no Estado de São Paulo. *Boletim do Departamento Nacional de Produção Mineral* **134**, 1–36 (1950).

40. Ghosh, S. C. Estheriids (fossil conchostraca) of Indian Gondwana. *Palaeontologia Indica* **54**, 1–146 (2011).

41. Mitchell, J. The fossil Estheridae of Australia. *Proceedings of the Linnean Society of New South Wales* **52**, 105–112 (1927).

42. Bock, W. Howellisaura, New Name for Howellites Bock. *J Paleontol* **27**, 759–759 (1953).
